# Biological roles of the RNA m^6^A modification and its implications in cancer

**DOI:** 10.1038/s12276-022-00897-8

**Published:** 2022-11-29

**Authors:** Juyeong Hong, Kexin Xu, Ji Hoon Lee

**Affiliations:** Department of Molecular Medicine, University of Texas Health Science Center at San Antonio, San Antonio, TX USA

**Keywords:** Oncogenes, Biochemistry

## Abstract

The *N*^6^-Methyladenosine (m^6^A) modification of RNA transcripts is the most prevalent and abundant internal modification in eukaryotic messenger RNAs (mRNAs) and plays diverse and important roles in normal biological processes. Extensive studies have indicated that dysregulated m^6^A modification and m^6^A-associated proteins play critical roles in tumorigenesis and cancer progression. However, m^6^A-mediated physiological consequences often lead to opposite outcomes in a biological context-dependent manner. Therefore, context-related complexity must be meaningfully considered to obtain a comprehensive understanding of RNA methylation. Recently, it has been reported that m^6^A-modified RNAs are closely related to the regulation of the DNA damage response and genomic integrity maintenance. Here, we present an overview of the current knowledge on the m^6^A modification and its function in human cancer, particularly in relation to the DNA damage response and genomic instability.

## Cancer: Call to explore common mRNA modification

Further investigations are needed to understand the consequences of a common messenger RNA modification in human cancers. N6-methyladenosine (m^6^A) modification is vital to many biological processes, including maintaining DNA repair responses and genomic stability. However, m^6^A modification and proteins recognising this modification are significantly dysregulated in cancers, leading Ji Hoon Lee at the University of Texas Health Science Center at San Antonio, USA, and co-workers to review current knowledge of the function of m^6^A. Reduced levels of m^6^A modification are linked to breast and lung cancer progression, while in liver cancer the modification appears to play different roles at different times, both promoting tumour growth and inhibiting tumour development pathways. Determining how m^6^A-modified RNA and associated proteins affect DNA damage responses and genomic integrity in cancer could provide valuable therapeutic strategies.

## Introduction

The RNA m^6^A modification, that is, the methylation of adenosine at the nitrogen-6 position in an RNA molecule, is considered an epitranscriptomic and posttranscriptional regulatory mark. Among more than 150 posttranscriptional chemical modifications on RNA molecules^1–3^, m^6^A is the most abundant internal modification of mRNAs and noncoding RNAs. Since the discovery of m^6^A^4,5^, development of m^6^A transcriptome-wide mapping technology based on next-generation sequencing (NGS) methods has been used to extensively study this modification, and many related studies have found that this RNA chemical modification exhibits biological significance^6,7^. Indeed, the m^6^A modification is closely associated with almost all aspects of RNA-related biological processes, including transcription, pre-mRNA splicing and processing, pri-microRNA (pri-miRNA) processing, nuclear export, translation, RNA stability and decay^7–20^. In addition to its role in RNA metabolic processes, the m^6^A modification is involved in other biological processes, such as transcriptional regulation, signal transduction, and the DNA damage response^21–26^. As the m^6^A modification and associated factors are significantly dysregulated in cancers, understanding their roles in tumorigenesis and cancer progression will provide in-depth insight into the development of new therapeutic strategies for cancer treatment. In this review, we describe the current understanding of m^6^A modification and its function in biological processes and cancers, particularly its contribution to the DNA damage response and genomic instability.

## The dynamics of the m^6^A modification and its molecular functions in RNA metabolism

**Fig. 1:**
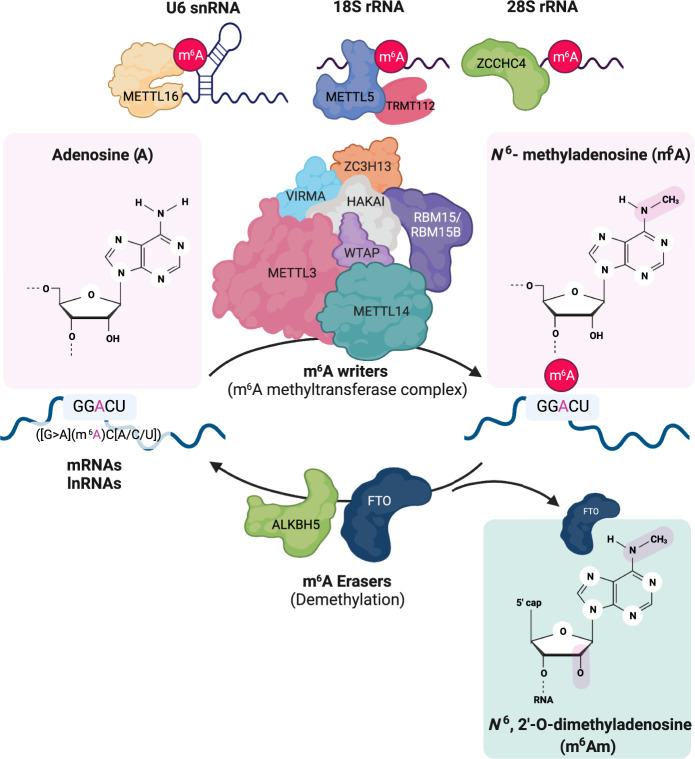
An overview of *N*^6^-methyladenosine (m^6^A) and m^6^A-associated enzymes. A m^6^A methyltransferase complex is composed of core m^6^A writer components (METTL3, METTL14, and WTAP) and regulatory proteins (VIRMA, RBM15/RBM15B, HAKAI, and ZC3H13). The m^6^A writer adds a methyl group to the *N*^6^-position of adenosine within the consensus sequence ([G > A](m^6^A)C[A/C/U]) of an RNA transcript (mRNA, lncRNA, and so on). Another methyltransferase comprising METTL16, METTL5 and ZCCHC4 specifically methylates snRNA, rRNA and a few noncoding RNAs. WTAP is an adapter in the regulation of the nuclear localization and activity of methyltransferase with regulatory proteins. The m^6^A erasers are ALKBH5 and FTO. ALKBH5 is a primary m^6^A demethylase that removes the methyl group from N^6^ adenosine from target mRNAs. FTO demethylates both internal m^6^A and *N*^6^,2′-O-dimethyladenosine in the 5′ cap (m^6^Am). This image was created with BioRender (https://biorender.com/).

The m^6^A modification is dynamically deposited and removed by m^6^A methyltransferase complexes (m^6^A writers) and demethylases (m^6^A erasers), respectively (Fig. 1). As the core subunit of the m^6^A methyltransferase complex, METTL3 and METTL14 form a heterodimer and recognize the consensus sequence motif [G > A](m^6^A)C[A/C/U], which is preferentially located near stop codons, 3′ untranslated regions (UTRs), and long internal exons^6,7,17,18^. The m^6^A methyltransferase activity of the METTL3-METTL14 heterodimeric complex is modulated by regulatory proteins, including WTAP, VIRMA, RBM15/15B, ZC3H13 and HAKAI;^19–21,27^ these factors are required for nuclear localization as well as the recruitment of the m^6^A methyltransferase complex to target RNA substrates. The expression levels or activities of the components of the m^6^A methyltransferase complex can alter the overall level of m^6^A in cells, and this change significantly affects transcriptome-wide landscape and biological functions. Although extensive studies have identified components of the m^6^A methyltransferase complex, silencing each component of the current m^6^A methyltransferase complex via RNA interference (RNAi) or gene knockout (KO) partially reduced the level of m^6^A in cells but did not abrogate it. Therefore, future studies need to be focused on investigating uncharacterized components of the m^6^A methyltransferase complex or enzymes that regulate the abundance of the m^6^A modification in cells.

One of the putative human m^6^A writer proteins, METTL16, is critical for methylating a few transcripts, such as the *U6* small nuclear RNA (snRNA), *MALAT1, XIST*, and the pre-mRNA *MAT2A*^28–32^. However, it has been recently reported that METTL16 preferentially localized to the cytosol and methylated more than 334 mRNA transcripts. In addition, *METTL16* KO caused a significant reduction in the rate of m^6^A deposition on target nascent RNAs compared to poly(A) RNAs. Interestingly, in addition to the previously proposed role involved in RNA splicing, METTL16 interaction with eukaryotic initiation factor 3a/b (eIF3a/b) and ribosomal RNAs (rRNAs) promotes ribosome assembly, resulting in enhanced translation of more than 4000 target mRNAs^33^. However, this translational regulation by METTL16 is neither methyltransferase activity-dependent nor m^6^A dependent.

Two other putative m^6^A writer proteins are METTL5 and ZCCHC4, which are critical for the m^6^A modification of 18 S rRNA and 28 S rRNA, respectively^34,35^. METTL5, not ZCCHC4, forms a heterodimeric complex with TRMT112, a methyltransferase activator^34^ (Fig. 1). Their biological function on 18 S and 28 S rRNAs remains unknown because knocking out *Mettl5* or *Zcchc4* negligibly affected human colon cancer HCT116 cell growth or mature rRNA processing and production^34^. However, a study showed that the proliferation of *ZCCHC4* KO HepG2 cells, derived from the liver tissue of a patient with hepatocellular carcinoma (HCC), was significantly inhibited, which was consistent with the defective translation of specific mRNAs in regulating tumorigenesis^34^. This phenotypical discrepancy of *ZCCHC4* KO might be cell-type specific or context dependent. Nevertheless, METTL5 and ZCCHC4 clearly methylate 18 S and 28 S rRNAs. Thus, the biological significance of METTL5- or ZCCHC4-mediated rRNA methylation in tumorigenesis should be addressed in different contexts.

**Fig. 2:**
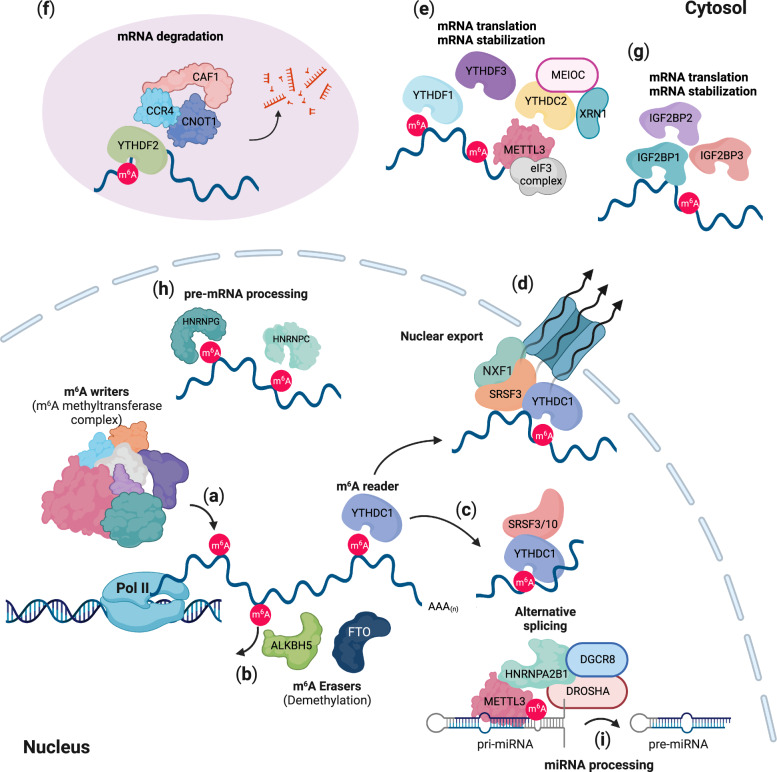
Cellular and molecular functions of *N*^6^-methyladenosine (m^6^A). **a** The m^6^A modification occurs cotranscriptionally via an m^6^A methyltransferase complex (an m^6^A writer). **b** ALKBH5 and FTO dynamically demethylate N^6^ adenosine in target RNAs. m^6^A readers (YTHDC1-2, YTHDF1-3, IGF2BP1-3, HNRNPC/G, and HNRNPA2B1) determine the fate of m^6^A-modified RNAs. **c** YTHDC1, a nuclear m^6^A reader, interacts with SRSF3/10 and regulates alternative splicing. **d** YTHDC1 also controls nuclear export mediated through SRSF3 and NXF1. **e** YTHDC2, YTHDF1, and YTHDF3 are involved in mRNA stabilization and translation. **f** YTHDF2 interacts with the mRNA decay machinery and the CCR4-NOT complex, leading to mRNA degradation. **g** IGF2BP1-3 regulate the stabilization and translation of mRNAs. **h** HNRNPC and HNRNPG modulate pre-mRNA processing. **i** HNRNPA2B1 recruits DROSHA-DGCR8 to pri-miRNA for miRNA processing. This image was created with BioRender (https://biorender.com/).

Increasing evidence indicates that the m^6^A modification is deposited cotranscriptionally on nascent transcripts^22,36–39^ (Fig. 2a). In acute myeloid leukemia (AML), CAATT-box binding protein (CEBPZ) directs METTL3 at the transcriptional start site (TSS) in a METTL14-independent manner^22^. METTL3 recruitment by CEBPZ promotes the m^6^A modification of target mRNA transcripts and enhances their translation. In mouse embryonic stem cells (mESCs), m^6^A deposition by Mettl3 occurs cotranscriptionally at Mettl3-bound chromatin regions, as indicated by both the genomic binding of Mettl3 and m^6^A modification are mainly enriched in the 3’UTR^36^. Moreover, m^6^A modification depends upon the transcriptional dynamics of RNA polymerase II^38^. Attenuated transcriptional activity of RNA polymerase II induced an increase in m^6^A modification abundance mediated through the physical interaction between RNAPII and METTL3, resulting in inefficient translation. These studies suggest that the m^6^A modification is, at least, a cotranscriptional event and is one of the factors linking transcription to translation. However, H3 trimethylation at Lys36 (H3K36me3), is associated with transcript elongation, which recruits the m^6^A methyltransferase complex to chromatin through the physical interaction between H3K36me3 andMETTL14 to deposit m^6^A cotranscriptionally on nascent transcripts^39^. Furthermore, several study groups have independently shown that nascent RNAs labeled with 4-thiouridine (4SU) in a short time (5–20 min) were deposited with m^6^A^25^, and chromatin-associated regulatory RNAs (carRNAs) were highly modified with m^6^A marks, revealing it to be among the major substrates for the methyltransferase of m^6^A modification^40,41^. Overall, the data obtained to date suggest that m^6^A deposition occurs cotranscriptionally on nascent- and chromatin-associated RNA molecules via chromatin association with the m^6^A methyltransferase complex.

The identification of two m^6^A demethylases, namely, the m^6^A erasers α-ketoglutarate-dependent dioxygenase alk B homolog 5 (ALKBH5) and fat mass and obesity-associated protein (FTO), has suggested that m^6^A deposition is dynamically reversible^23,24^. ALKBH5 selectively removes the methyl group from *N*^6^-adenosine of target mRNAs, whereas FTO demethylates both internal m^6^A marks and *N*^6^,2′-O-dimethyladenosine in the 5′ cap (m^6^Am)^10^, suggesting that ALKBH5 is a primary m^6^A demethylase (Fig. 2b). Interestingly, notable phenotypes in mammalian development have indicated that these two m^6^A demethylases play a broad regulatory role in developmental processes^23,24^. *Fto*-KO mice display partial embryonic lethality, postnatal growth retardation, and increased postnatal lethality^41–45^. Recently, it has been reported that RNA transcribed from long-interspersed element 1 (LINE1) is a physiological substrate of FTO that regulates the chromatin state in mammalian tissues and during development^41^*. Alkbh5* KO led to impaired fertility through a dysregulated splicing process in sperm development^24,46^, although *Alkbh5*-KO mice were viable and reached adulthood. Indeed, ALKBH5 localizes to nuclear speckles (also known as interchromatin granule clusters) and nuclear domains enriched in pre-mRNA splicing factors^24^. Deficient Alkbh5 leads to high levels of m^6^A deposition on spermatogenesis-associated mRNAs and induces aberrant RNA splicing in nuclear speckles, resulting in the abrogation of fertility. Taken together, the data show that, along with the m^6^A methyltransferase complex, the identification of two m^6^A demethylases indicates the reversible and dynamic m^6^A modification of mRNAs, which plays critical roles in fundamental biological processes, adding a layer to posttranscriptional regulatory mechanisms.

The m^6^A modification determines the fate of RNAs via selective m^6^A-recognizing factors (m^6^A readers). The YTH domain-containing proteins YTHDC1-2 (YTHDC1 and YTHDC2) and YTHDF1-3 (YTHDF1, YTHDF2, and YTHDF3) are direct m^6^A readers that modulate the fate of m^6^A-modified RNAs in cells. First, YTHDC1 binds m^6^A-modified target RNAs and regulates RNA splicing, nuclear export, exosome-mediated RNA decay and stability of carRNAs in the nucleus^14,25,40,47–51^ (Fig. 2c, d). YTHDC2 also regulates the stability of m^6^A-modified RNAs and promotes translation efficiency in the cytoplasm^52,53^ (Fig. 2e). YTHDF1-3 bind m^6^A-modified mRNAs and facilitate cytosolic mRNA decay^9,54,55^ (Fig. 2e, f). In particular, YTHDF1 and YTHDF3 enhance the translation of m^6^A-modified mRNAs with translation initiation complexes^55,56^ (Fig. 2e). In addition to YTH domain-containing proteins, insulin-like growth factor 2 mRNA-binding proteins, IGF2BP1-3, recognize the m^6^A mark on mRNAs and thus regulate their stability and translation^57,58^ (Fig. 2g). Finally, heterogeneous nuclear ribonucleoproteins (HNRNPs), namely, hnRNPA2B1, hnRNPG, and hnRNPC, are potential m^6^A readers^59–61^ (Fig. 2h, i). However, since they bind to m^6^A-modified RNAs and change the structure of target RNAs, they seem to play roles as “m^6^A switches” not as direct m^6^A readers^60,61^ (Fig. 2h). Nevertheless, hnRNPA2B1 modulates the processing of m^6^A-modified primary miRNAs (pri-miRNAs) through the recruitment of DGCR8, a component of the microprocessor complex, and regulates the alternative splicing process^59^ (Fig. 2i).

## Cellular and molecular functions of the m^6^A modification

### mRNA instability by the m^6^A modification

One of the best characterized functions of m^6^A modification causes the destabilization of m^6^A-modified mRNAs^8,9,62^. Although all YTHDF1-3 proteins contribute to the destabilization of m^6^A on target mRNAs, recent studies have implied that YTHDF2 is the major m^6^A reader involved in the decay of m^6^A-modified RNAs^9,63,64^ (Fig. 2e, f). Increasing evidence has revealed that YTHDF2 is required for directing mRNAs to processing bodies (P-bodies), where mRNA decay-associated proteins accumulate^9,54,65,66^ (Fig. 2f). All three YTHDF1-3 proteins facilitate phase-separation with m^6^A-modified mRNAs to induce the accumulation of transcripts at P-bodies, stress granules and neuronal RNA granules^54^. Independent of P-bodies, YTHDF2 directly interacts with CNOT1 and recruits the CCR4/NOT deadenylase complex to m^6^A-modified mRNAs, leading to the deadenylation and degradation of mRNAs^64^. Furthermore, YTHDF2 associates with RNase P/MRP, an endoribonuclease, through direct interaction with heat-responsive protein 12 (HRSP12)^63^. The depletion of any of these three proteins abrogated m^6^A-mediated degradation of mRNAs, suggesting that YTHDF2 recognition of m^6^A-modified mRNAs mediates mRNA decay mediated via HRSP12 and RNase P/MRP. A transcriptome-wide analyses of HRSP12-binding sites and cleavage sites of RNase P/MRP further supported the finding that HRSP12 binds upstream of YTHDF2-binding sites and that RNase P/MRP endoribonucleolytically cleaves downstream YTHDF2-binding sites within target m^6^A-modified mRNAs, demonstrating that the m^6^A modification promotes the degradation of target mRNAs through the recruitment of RNase P/MRP mediated via HRSP12.

### Alternative splicing by the m^6^A modification

Several lines of evidence show the role played by the m^6^A modification in mRNA splicing. Previous reports showed that the overall levels of m^6^A on mRNAs were significantly enriched in the early onset of embryogenesis in *Drosophila* and were rapidly decreased during embryogenesis. Ime4, a *Drosophila* METTL3 homolog, regulates the female-specific splicing of the Sex-lethal (*Sxl*) gene^67,68^. The m^6^A demethylases FTO and ALKBH5 are also involved in splicing machinery, regulating alternative splicing of long 3’UTRs containing pre-mRNAs or a subset of adipogenesis-associated mRNAs, respectively^24,46,69^. Transcriptome-wide mapping of RNAs bound by FTO showed significant overlap with previously reported m^6^A locations within intronic regions of pre-mRNAs, and depletion of FTO led to the inclusion of alternatively spliced exons^70^. ALKBH5 also regulates the proper splicing of longer 3’UTR transcripts, particularly in mitotic and meiotic male germ cells^46^. Even though m^6^A may affect the splicing process in only a subset of genes, these m^6^A-mediated splicing events might be functionally important. Although the abundance of m^6^A marks is related to modulated alternative splicing, the m^6^A reader protein YTHDC1 interacts with splicing regulators, including SAM68, SC35, SRSF1 and SRSF3, suggesting that the m^6^A reader plays a role in mRNA splicing^47,48,71,72^ (Fig. 2c). However, it remains unclear whether YTHDC1 activity is coordinated with these splicing regulators in a m^6^A-dependent or m^6^A-independent manner.

### Nuclear export mediated via the m^6^A modification

The m^6^A modification influences the nuclear export of m^6^A-modified mRNA^14,15,24^. ALKBH5 loss induced the nuclear accumulation of m^6^A-modified mRNAs, suggesting that the m^6^A mark mediated mRNA export^24^. Another study demonstrated that YTHDC1 was involved in the nuclear export of m^6^A-modified mRNAs via its interaction with splicing factor SRSF3 and nuclear RNA export factor 1 (NXF1)^14^ (Fig. 2d). These results are supported by the interaction exhibited between the m^6^A-methyltransferase complex YTHDC1 and the TREX (TRanscription-EXport) mRNA export complex^15^. The nuclear export of mRNAs is cotranscriptionally coupled with the capping, splicing and 3′ end processing of primary transcripts^73^. However, although the m^6^A modification of pre-mRNAs has been associated with the splicing process, it has been recently reported that the role of m^6^A modification in pre-mRNA splicing is limited to a small number of pre-mRNA groups^37^, suggesting that the m^6^A-mediated pre-mRNA splicing process is not closely related to the nuclear export process. The TREX subunits ALY/REF and THOC5 make contact with NXF1, expose the RNA-binding domain of NXF1, and allow the interaction between NXF1 and mRNA^74^. However, it remains unclear exactly how the m^6^A mark regulates nuclear mRNA export between the nucleus and cytoplasm. Nevertheless, these studies suggest a role for m^6^A in nuclear export.

### RNA translation mediated via the m^6^A modification

Myriad studies have demonstrated that m^6^A modification regulates the efficient translation of m^6^A-modified mRNAs. Some YTH domain-containing m^6^A readers, including YTHDF1, YTHDF3 and YTHDC2, have been reported to enhance the translation of m^6^A-modified mRNAs^55,56,75^ (Fig. 2e). In particular, YTHDF1 promotes the translation of m^6^A-modified mRNAs through its interaction with the eukaryotic translation initiation factor eIF3 complex^56^. YTHDF1 binds to m^6^A sites that are located around a stop codon and in the 3′UTR and facilitates translation. However, it remains to be investigated how eIF3 regulates the translation of m^6^A-modified mRNAs, since eIF3 is recruited to the 5′UTR or upstream of the translation start site for translation initiation^76^. Another study reported that eIF3 binds directly to 5′UTR-m^6^A sites, leading to the recruitment of the ribosomal 43 S preinitiation complex^12^. Translation initiation by eIF3 binding to 5′UTR-m^6^A sites does not require the cap-binding factor eIF4E, suggesting cap-independent translation. In addition, most recent studies demonstrate that eIF3H at the 5′UTR directly binds to METTL3 at the 3′UTR-m^6^A sites, promoting mRNA circularization and increasing cap-dependent or cap-independent ribosome translation efficiency^77,78^. It is still unclear how METTL3 binds to m^6^A-modified mRNA and contributes to translation efficiency. Future studies should address the molecular details of how METTL3 and readers recognize m^6^A marks and how the m^6^A modification contributes to each step of translation.

## RNA m^6^A modifications in cancer

Although the current knowledge of the precise mechanism by which m^6^A modification regulates diverse biological processes remains to be further explored, an increasing number of studies examined the effects of m^6^A modification in various types of cancer. In this section, we summarize the recent findings of these studies with respect to the most common types of human cancer.

### Acute myeloid leukemia (AML)

AML is the most common hematopoietic malignant leukemia in adults. Recurring chromosomal aberration and genetic mutations as well as aberrant alteration of epigenetic modifications, including DNA methylation and histone modification, contribute to hematopoietic malignancies such as AML^79^. The m^6^A methyltransferase METTL3 is more abundant in AML cells than in CD34-positive stem and hematopoietic progenitor cells (HSPCs) and is required for the differentiation of AML cells^22,80^. METTL3 binds to promoters associated with the differentiation of AML in a METTL14-independent manner, leading to direct transcriptional activation. METTL3 is recruited by CEBPZ at a transcriptional start site, and promoter-bound METTL3 adds the m^6^A mark to a cognate mRNAs, enhancing its ribosomal translation^38^. A study revealed that METTL3 deposited the m^6^A mark to pro-oncogenes such as the MYC proto-oncogene (*c-MYC*), B-cell lymphoma 2 (*BCL2*), and phosphatase and tensin homolog (*PTEN*), activating phosphoinositide 3-kinase (PI3K) and protein kinase B (PKB) signaling pathways, which regulate cell differentiation and self-renewal^80^. METTL14, a key component of the m^6^A methyltransferase complex, is highly expressed in HSPCs and AML cells^81^. Although negatively regulated by SPI1, METTL14 is involved in the m^6^A modification of target mRNAs of *MYB* and *MYC*, promoting translation and inhibiting myeloid differentiation. Interestingly, FTO, a m^6^A eraser, also plays a critical oncogenic role in AML, promoting leukemic oncogene-mediated transformation and leukemogenesis by regulating target mRNAs, such as *ASB2* and *RARA* mRNAs, through the removal of methyl group from *N*^6^-adenosine^82^. Moreover, YTHDC1 and YTHDF2, m^6^A readers, play important roles in the survival and differentiation of AML cells^51,83^. YTHDC1 undergoes liquid‒liquid phase separation with m^6^A-modified mRNAs and forms nuclear YTHDC1-m^6^A condensates (nYACs)^51^. Abundant nYACs in AML cells protect m^6^A-modified mRNAs (i.e., *MYC* and others) from the polyA tail exosome targeting complex (PAXT) and exosome-associated RNA degradation^51,84^. YTHDF2 destabilizes m^6^A-modified mRNAs (i.e., tumor necrosis factor receptor Tnfrsf2 and others) that are associated with the function of self-renewing leukemic stem cells (LSCs), contributing to the initiation of AML^83^.

### Hepatocellular carcinoma (HCC)

Liver cancer is a highly progressive and the second most life-threatening tumor^85,86^. It comprises hepatocellular carcinoma (HCC) and intrahepatic cholangiocarcinoma (iCCA) in accordance with histological features^85,86^. Recent reports have shown that METTL3 is upregulated in human HCC, leading to m^6^A hypermethylation of the tumor suppressor SOCS2 (Suppressor Of Cytokine Signaling 2)^87^. The m^6^A reader protein YTHDF2-dependent RNA degradation pathway mediates the degradation of *SOCS2* mRNAs, suggesting that METTL3 represses the expression and stability of critical tumor suppressor genes at the posttranscriptional level. In addition, METTL3 regulates the expression of USP7 (Ubiquitin Specific Peptidase 7) through the m^6^A modification of its target mRNAs^88^. Upregulated USP7 expression resulted in an increase in oncogenic activities of HCC cells. However, YTHDF2 functioned as a tumor suppressor in HCC via destabilization of epidermal growth factor receptor (*EGFR*) mRNA^89^, leading to the inhibition of the ERK/MEK signaling pathway in HCC. These results suggest that m^6^A modification has a dual role in HCC by promoting HCC tumor progression or inhibiting oncogenic pathways. Thus, the role of m^6^A modification in HCC remains to be further explored.

### Glioblastoma (GBM)

Glioblastoma (GBM) is the most aggressive and common primary brain and central nervous system (CNS) malignancy in adults^90,91^. GBMs are characterized by heterogeneity; that is, they contain glioblastoma stem-like cell (GSC) populations with stem-like properties, contributing to tumor initiation and therapeutic resistance^92^. The METTL3 level is highly elevated in GSCs and is required for the maintenance of GSCs and the dedifferentiation of glioma cells through an m^6^A modification in the 3′ UTR of sex-determining region Y (SRY)-Box 2 (*SOX2*) mRNA^93^. The m^6^A modification of *SOX2* mRNAs and recruitment of human antigen R (HuR), which is also highly expressed in GBMs, are essential for SOX2 mRNA stabilization, which leads to the maintenance of GSCs. Most GBM cases are refractory to radiotherapy and the chemotherapy drug temozolomide (TMZ) via rapid DNA repair by O-6-methylguanine-DNA methyltransferase (MGMT)^94^. Both METTL3 and SOX2 regulate DNA repair genes and partially mediate m^6^A-dependent radioresistance^93^. Therefore, further studies are required to examine the potential of SOX2 as a predictor for the outcome of and benefit from TMZ chemotherapy, in addition to MGMT. Interestingly, ALKBH5 is highly expressed in GSCs and demethylates FOXM1 nascent transcripts with a long noncoding RNA antisense strand (FOXM1-AS), leading to enhanced FOXM1 expression, GSC proliferation, and tumorigenesis^95^. A study showed that m^6^A modification functions as a tumor suppressor for GSC self-renewal and tumorigenesis^96^. The abundance of m^6^A marks after METTL3 or METTL14 knockdown promoted the tumorigenesis of GSCs. In contrast, the inhibition of FTO by the ethyl ester form of meclofenamic acid (MA2) suppressed the progression of GSC-grafted tumors. Taken together, these results suggest that m^6^A-mediated tumor formation can lead to opposite results, even in the same context. Thus, future studies should address the context- or experimental condition-based comprehensive interpretation to understand the precise role of m^6^A modification in GBMs.

### Breast cancer

Breast cancer is the most frequently cancer diagnosed in women worldwide^97^. Breast cancer is heterogeneous and classified by the expression of hormone receptors (estrogen receptor and progesterone receptor) and human epidermal growth factor receptor 2 (HER2)^98^. The molecular subtypes of breast cancer are luminal A, luminal B, HER2-positive, and basal-like triple-negative breast cancer (TNBC), which are considered to show similar clinical behaviors prior to the treatment of breast cancer. In breast cancer, increasing evidence has shown that the m^6^A modification is critical for tumorigenesis and progression. Silencing of METTL14 and ALKBH5 significantly inhibited breast cancer cell growth and invasive activity^99^. METTL14 and ALKBH5 regulate m^6^A-modified mRNAs involved in the cell cycle, the epithelial-mesenchymal transition (EMT), and angiogenesis through the HuR-mediated stabilization of target mRNAs. In particular, a study demonstrated the specific role of m^6^A modification in regulating the TGFβ signaling pathway in the tumorigenesis of breast cancer. In contrast, another study showed that METTL14 overexpression increased the abundance of m^6^A marks and inhibited oncogenic activity^100^. In TNBC, a lower level of METTL3 and a higher level of FTO were associated with poor prognosis, indicating that a lower m^6^A mark level contributes to the progression of TNBC^101^. In addition, under hypoxic stress conditions, elevated ALKBH5 reduces the abundance of the m^6^A mark on pluripotency marker *NANOG* and *KLF4* mRNAs, leading to increased NANOG and KLF4 expression and an enhanced breast cancer stem cell phenotype^102^.

### Lung cancer

Lung cancer is one of the most common cancers in the world, and small-cell lung cancers (SCLCs) and non-small cell lung cancer (NSCLC) are the two major histologic subtypes^103,104^. Despite our understanding of the biology of this disease and mechanisms of lung tumor progression, the overall cure and survival rates for lung cancer patients remain very low, particularly for patients with metastatic disease. Increasing evidence indicates that METTL3 is highly expressed in NSCLC cells and related to oncogenic activity in lung cancer^77,78,105–107^. Although METTL3 is localized mainly in the nucleus, one study demonstrated that cytosolic METTL3 functioned as a m^6^A reader that bound to a 3′-UTR near a stop codon. m^6^A-modified mRNA-bound METTL3 directly interacted with eIF3h in eIF3 complex, facilitating mRNA looping to induce the recycling of polyribosomes^77,78^. Transcriptome-wide analyses demonstrated that METTL3 regulated a large subset of oncogenic mRNAs through the METTL3-eIF3h axis without affecting mRNA abundance, facilitating oncogenic activity^77,78^. In addition, several groups supported findings showing that downregulation of METTL3 expression levels through miR-600, miR-33a or RNAi clearly inhibited the oncogenic activities of lung cancer cells^105–107^. miR-600 and miR-33a bound to the 3′-UTR of *METTL3* mRNAs, leading to the degradation of *METTL3* mRNAs and subsequently inducing apoptosis in lung cancer cells. The level of METTL3 was also controlled posttranslationally. Indeed, METTL3 is SUMOylated by small ubiquitin-related modifier 1 (SUMO1), leading to a reduction in m^6^A methyltransferase activity. Thus, SUMOylation of METTL3 decreases the global cellular abundance of the m^6^A mark and subsequently alters the transcriptome of m^6^A-modified RNAs in cells, facilitating the development of NSCLC.

## The m^6^A modification and genomic instability

**Fig. 3:**
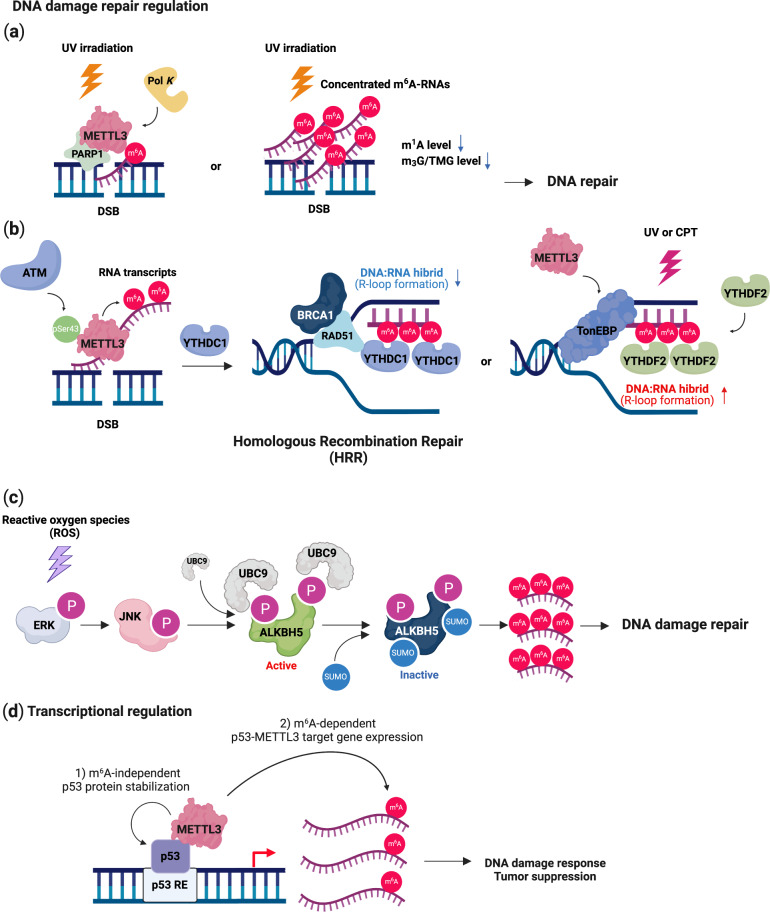
DNA damage response and repair by *N*^6^-methyladenosine (m^6^A). DNA damage response and repair. **a** Upon genotoxic stress, ATM phosphorylates METTL3, which binds to a DNA lesion, and m^6^A-modified RNAs direct PARP1 and Pol K for nucleotide excision repair (NER). **b** The m^6^A-modified RNAs are recognized by YTHDC1 or YTHDF2, which recruits RAD51 to the damaged region for homologous recombination repair (HRR). **c**. Reactive oxygen species (ROS) activate the ERK-JNK pathway, which phosphorylates ALKBH5. UBC9 binds to phosphorylated ALKBH5, inducing its SUMOylation. Inhibition of the demethylase activity of ALKBH5 by SUMOylation increases the level of m^6^A-modified RNAs that are related to DNA damage repair. Transcriptional regulation of DNA damage response and repair. **d** METTL3 stabilizes the p53 protein in a m^6^A-independent manner. METTL3 deposits an m^6^A mark on p53 target mRNAs for regulating the DNA damage response and tumor suppression. This image was created with BioRender (https://biorender.com/).

Several studies have found that METTL3-mediated m^6^A modification plays a critical role in the DNA damage response (DDR) to regulate the DNA repair pathway. RNA m^6^A modification rapidly occurs in ultraviolet (UV)-irradiated chromatin, indicating that METTL3 is specifically recruited to the UV-damaged chromatin region^108^ (Fig. 3a). This extensive recruitment of METTL3 depends on ADP-ribose polymerase 1 (PARP1). This DNA repair pathway may be mediated by trans-lesion DNA polymerase κ (Pol κ), which has been implicated in both nucleotide excision repair (NER) and trans-lesion synthesis (TLS)^108–110^. The m^6^A-mediated recruitment of Pol κ differentially regulates the UV-induced DNA damage response mediated by the canonical NER pathway and the Rad18/PCNA-regulated TLS pathway. However, the precise role of m^6^A modification and Pol κ in the UV damage response remains to be further investigated. Another study supported these findings by showing that nucleoplasmic fractions of m^6^A RNAs were immediately concentrated in UV-irradiated DNA lesions without affecting the level of METTL3, METTL14 or FTO^111^ (Fig. 3a). The authors showed that UV radiation reduced the levels of 2,2,7-methylguanosine (m_3_G/TMG) and *N*^1^-methyladenosine (m^1^A) in RNA as results of DNA damage. These results suggest that METTL3 rapidly localizes to UV-irradiated genomic regions and methylates RNAs and that these concentrated m^6^A-modified RNAs regulate the downstream DNA damage repair pathway to promote cell survival.

METTL3-mediated m^6^A modification also mediates homologous recombination (HR)-mediated double-strand DNA (dsDNA) break repair^112^ (Fig. 3b). METTL3 is phosphorylated at Ser43 by ataxia telangiectasia mutated (ATM) in response to double-strand breaks (DSBs). Phosphorylated METTL3 can be localized at DSB regions, leading to the m^6^A modification of nascent RNAs derived from damaged chromatin regions. These m^6^A-modified RNAs are recognized by YTHDC1, resulting in the formation of DNA‒RNA hybrids at DSBs. Subsequently, the formation of DNA‒RNA hybrids induces the recruitment of repair-related proteins, including RAD51 and BRCA1, to promote or HR-mediated repair, preventing genomic instability^113,114^. Depletion of METTL3 significantly enhanced the sensitivity of cancer cells and murine xenograft models to DNA damage-based therapies, such as chemotherapy drugs or radiation. Furthermore, a higher level of METTL3 predicted a poor survival probability for head and neck squamous carcinoma (HNSC) patients who had been treated with cisplatin or radiation. These results suggest that m^6^A modification in DSB repair is a potential target for cancer therapy. However, since METTL3 can increase the efficiency of DSB repair, it may also contribute to drug resistance in DNA damage-based treatment^93,112^.

RNA m^6^A modification also occurs in the majority of DNA‒RNA hybrids (R-loops) in human pluripotent stem cells^115^ (Fig. 3b). The m^6^A modification of RNA in R-loops is increased during the G2/M phase and disappears in the G0/G1 phase of the cell cycle, indicating cell cycle-dependent regulation of this modification. YTHDF2 binds to m^6^A-modified RNAs in R-loops, leading to the degradation of RNAs and the reduction in the number of R-loops. Inhibition of METTL3 or YTHDF2 results in the accumulation of R-loops and γH2AX, a DSB marker, and subsequently induces cell growth retardation. Thus, the regulation of METTL3- and YTHDF2-mediated RNA–DNA hybrids may represent a critical process in preventing genomic instability caused by the accumulation of cotranscriptional R-loops during mitosis. Furthermore, m^6^A can also resolve R-loops induced by DNA damage via UV or camptothecin (CPT) through tonicity-responsive enhancer-binding protein (TonEBP)^116^. TonEBP directly binds to R-loops and recruits METTL3, leading to m^6^A modification on an RNA strand of the R-loop. TonEBP also recruits RNase H1 to resolve R-loops. However, in different studies and cellular contexts, the m^6^A modification promoted R-loop formation to facilitate transcription termination^117^, suggesting context-dependent regulation. Nevertheless, the studies demonstrate that the m^6^A modification plays a critical role in regulating R-loops, the DNA damage response, and genomic stability.

It has been reported that FTO is important for the maintenance of bone mass and functions because it protects osteoblasts from genotoxic damage^45^. Previously, large-scale genome-wide association studies (GWAS) showed that FTO was closely linked to obesity and body composition in multiple human populations^118–120^. FTO specifically removes a methyl group from the N^6^-adenoside on the mRNAs of DNA repair genes (e.g. *Hspa1*, *Cdk9*, *Kdm2a*, and *Ube2v1*), leading to increased mRNA stability^45^. Subsequently, upregulation of *Hspa1a* and DNA repair genes protects osteoblasts from genotoxic agent (UV and H_2_O_2_)-mediated apoptosis. In addition, FTO protects osteoblasts from genotoxic damage induced by metabolic stress caused by the loss of Fto, which exacerbated osteoblast DNA damage in mice fed a high-fat diet. ALKBH5 also plays a role in the regulation of the DNA damage response and apoptosis mediated via reactive oxygen species (ROS)^121^ (Fig. 3c). ROS activate the ERK/JNK signaling pathway, which phosphorylates ALKBH5 at Ser87 and Ser321. Phosphorylated ALKBH5 interacts with UBC9, a SUMO E2 conjugating enzyme, leading to SUMOylation of ALKBH5 at Lys86 and Lys321. The enzymatic activity of SUMOylated ALKBH5 was thus inhibited, and then, the abundance of m^6^A on the mRNA of DNA repair genes increased, protecting cells from ROS-induced DNA damage response.

The m^6^A mark engages in crosstalk with the transcription factor, p53, and regulates the p53-mediated transcriptomic program induced by DNA damage stimuli^122^ (Fig. 3d). METTL3 has been identified as a p53-interacting partner after treatment with the DSB inducer doxorubicin. METTL3 stabilizes the p53 protein in a m^6^A-independent manner. However, METTL3 deposited the m^6^A mark on p53-targeted mRNAs to regulate the DNA damage response and tumor suppression only in the presence of an intact p53 protein. Therefore, further investigation should address whether RNA is dispensable for the direct interaction between METTL3 and p53 since p53 can bind to other RNA species^123–125^.

**Fig. 4:**
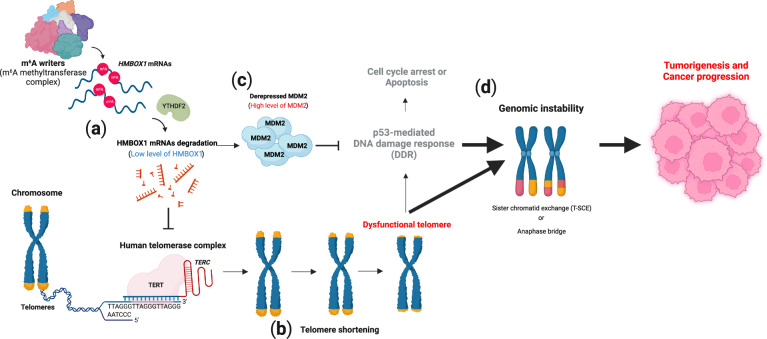
Telomere shortening-driven genomic instability by *N*^6^-methyladenosine (m^6^A) and tumorigenesis. **a** *N*^6^-methyladenosine (m^6^A) of *HMBOX1* mRNA induces YTHDF2-mediated mRNA degradation. **b** A reduction in HMBOX1 gradually shortens telomeres. **c** Although shortened telomeres induce a dysfunctional telomere-driven DNA damage response (DDR), derepressed MDM2 inactivates the p53-mediated pathway, which is involved in cell cycle arrest or apoptosis. **d** Genomic instability mediated through sister chromatid exchange (T-SCE) or an anaphase bridge promotes tumorigenesis and cancer progression. This image was created with BioRender (https://biorender.com/).

The m^6^A RNA modification is involved in the regulation of telomere length and genomic integrity in human cancers^26^ (Fig. 4). Telomeres are specialized structures at the ends of mammalian linear chromosomes and consist of tandem TTAGGG DNA nucleotide repeats^126^. Shelterin, a protein complex that binds to single- or double-strand telomeres, protects telomeres from being inappropriately recognized as damaged DNA^127,128^. In human cancer cells, telomerase, which consists of the catalytic subunit TERT and RNA template *TERC*, adds TTAGGG repeats during every cell division, preventing gradual telomere shortening due to the end replication problem of semiconservative DNA replication^129^. In normal somatic cells, *TERT* is transcriptionally repressed, and therefore, telomere length is gradually shortened with defective telomerase activity and progressive cell divisions, leading to a shortened-telomere-driven crisis point^129^. A shortened telomere crisis is closely linked to the genomic alterations found in cancer-relevant genomes^130^. Homeobox-Containing 1 (HMBOX1, also known as HOT1 or TAH1) is a mammalian telomere-binding protein that is involved in the recruitment of active telomerase and is required for telomere maintenance in the alternative lengthening of telomeres (ALT) in cancer cells^131,132^. Notably, *HMBOX1* mRNA has been identified as a *de novo* target for m^6^A modification in cancer cells^26^. The authors of this study found that m^6^A marks in the 3′ UTR of *HMBOX1* mRNA facilitated its degradation through YTHDF2 (Fig. 4a). In line with the function of HMBOX1 in the recruitment of telomerase to telomeres, downregulation of HMBOX1 mediated by the overexpression of METTL3 failed to maintain telomere length, as indicated by the defective recruitment of telomerase to telomeres^26,131^ (Fig. 4b). A previous report showed that HMBOX1 functions as a transcriptional repressor^133^. HMBOX1 suppresses the expression of MDM2 and is essential for the competency of p53 signaling^26^ (Fig. 4c). METTL3 upregulation in human cancer cells leads to shortened telomere-driven telomere dysfunction and inactivation of the p53-dependent DNA damage response pathway through MDM2 derepression^26^ (Fig. 4b, c). In the cancer-relevant genome, this coordinating environment might contribute to various types of telomere-associated chromosomal aberrations (e.g., translocations, amplifications, and deletions), enhancing the tumorigenicity and aggressiveness of cancer cells (Fig. 4d). Taken together, these results suggest an unexpected regulatory role for m^6^A marks in telomere biology and genome integrity.

## Concluding remarks and future directions

The review discusses our current understanding of the multifaceted roles of the m^6^A modification in regulating RNA metabolic processes and related biological processes, including the DNA damage response and genomic instability. The role of m^6^A modification and m^6^A-associated proteins is cell- or disease-context dependent. Because of the significant role played by m^6^A in a variety of biological and physiological processes, context-dependent coordinated action among m^6^A-associated proteins determines the outcomes of m^6^A modification. The molecular details of the crosstalk between m^6^A modification and m^6^A-associated proteins and how this crosstalk affects diverse biological processes still need to be investigated. In particular, understanding how m^6^A modification and its associated proteins modulate DNA damage responses to maintain genomic integrity may lead to a new therapeutic strategy in cancer.
